# Exosomal microRNA from Plasma in Patients with Pseudoexfoliation Glaucoma of Korea

**DOI:** 10.3390/ijms26094244

**Published:** 2025-04-29

**Authors:** Hyo Jung An, Dae Hyun Song, Changwon Kee, Hyun-kyung Cho

**Affiliations:** 1 Department of Pathology, Gyeongsang National University Changwon Hospital, School of Medicine, Gyeongsang National University, Changwon 51472, Republic of Korea; ariel2020@naver.com (H.J.A.); golgy@hanmail.net (D.H.S.); 2 Institute of Medical Science, School of Medicine, Gyeongsang National University, Jinju 52727, Republic of Korea; 3 Department of Ophthalmology, Samsung Medical Center, School of Medicine, Sungkyunkwan University, Seoul 06351, Republic of Korea; ckee@skku.edu; 4 Department of Ophthalmology, Gyeongsang National University Changwon Hospital, School of Medicine, Gyeongsang National University, Changwon 51472, Republic of Korea

**Keywords:** exosome, exosomal microRNA, glaucoma, microRNA, pseudoexfoliation glaucoma, RNA sequencing

## Abstract

This study aimed to determine the microRNA (miRNA) profile extracted from exosomes in plasma samples in pseudoexfoliation (PEX) glaucoma patients compared to controls. A blood sample (10 mL) was obtained after acquiring written informed consent. Exosome was extracted from each plasma sample using an Exoquick-TC kit. RNA sequencing was performed for each exosome sample. A bioinformatics study was conducted for miRNA-related pathways and targets. A total of 14 Korean subjects (7 with PEX glaucoma; 7 age-matched controls) were involved in the final study. In exosomes of PEX glaucoma participants, 330 mature miRNAs were detected. Among these, three miRNAs were significantly upregulated, including hsa-miR-92b-5p (fold change: 24.68), hsa-miR-744-5p (fold change: 2.49), and hsa-miR-148b-3p (fold change: 3.96). Sixty-six miRNAs were significantly downregulated in PEX glaucoma patients compared to the controls (all *p* < 0.05). These significantly altered miRNAs (both upregulated and downregulated) were associated with the gene ontology (GO) category of neurogenesis (9.41%), which accounted for the largest proportion. The expression of exosomal microRNAs in plasma was significantly different between PEX glaucoma patients and the controls. This suggests their possible roles in the pathogenic mechanism and a good diagnostic marker for PEX glaucoma.

## 1. Introduction

Glaucoma is a major cause of visual impairment globally and may ultimately lead to blindness [1,2]. The estimated worldwide prevalence of primary open-angle glaucoma (POAG) is 68.56 million individuals (95% CI: 59.99–79.98) [1], with Asian people comprising approximately 53.81% of these cases [1]. As a neurodegenerative disorder, glaucoma is defined by the histopathological degeneration of retinal ganglion cells (RGCs) [3].

Pseudoexfoliation (PEX) syndrome, also referred to as PEX glaucoma, represents one of the most common causes of secondary glaucoma [4]. PEX syndrome is an age-related disorder associated with alterations in the extracellular matrix (ECM), leading to the progressive production and accumulation of distinctive white, tiny fibrillary depositions in various extra- and intraocular tissues [4]. Compared to POAG, PEX glaucoma typically exhibits higher intraocular pressure (IOP), reduced responsiveness to hypotensive anti-glaucoma medications, and a more aggressive disease course [4]. The precise etiology of PEX syndrome remains unclear; however, genetic factors have been hypothesized to contribute to its pathogenesis [5,6,7]. Genetic variability among individuals may account for the differences in the global prevalence of PEX syndrome and PEX-associated glaucoma [5,6,7].

Exosomes are small extracellular vesicles, ranging in diameter from 30 to 150 nm, encapsulated by a lipid bilayer. They serve as carriers of molecular cargo, including mRNAs, proteins, and miRNAs, thereby facilitating intercellular communication [8]. Beyond their role as messengers, exosomes are also considered intrinsically therapeutic nanoparticles (ITNPs) with bioactive properties that offer promising diagnostic and therapeutic potential [9]. Exosomes are secreted by various cell types and have been identified in multiple biological fluids, such as urine, blood plasma, and aqueous humor (AH) [10,11,12]. Their primary function is to mediate intercellular signaling through the transmission of bioactive molecules, including RNAs and proteins [11,13,14]. Due to their role in cellular communication, exosomes hold promise as biomarkers and therapeutic agents [11,13,14]. Their involvement in ocular physiology and pathology, particularly in optic neuropathies such as glaucoma, has been reported in multiple studies, including review articles [15,16].

In our previous study, we identified differential microRNA expression in the aqueous humor (AH) of patients with normal-tension glaucoma (NTG) and pseudoexfoliation (PEX) glaucoma, revealing significant variations between the two groups [17]. We have also reported that the microRNA expression profile of NTG patients is significantly different from that of control patients [18]. Since microRNA expression is significantly different among different types of glaucoma, investigating the profile of the microRNA of exosome in a specific type of glaucoma such as PEX glaucoma might be significantly meaningful. In a separate study, we quantitatively analyzed exosomes in the aqueous humor (AH) of patients with pseudoexfoliation (PEX) glaucoma compared to control subjects within a Korean population [19]. Utilizing an advanced detection platform based on a tangential flow filtration system and a single-particle interferometric reflectance imaging sensor, we observed a significantly increased exosome count in the AH of PEX glaucoma patients relative to the controls. These findings suggest a potential role for exosomes in the pathogenesis of PEX glaucoma and further underscore the involvement of microRNAs within exosomes as potential biomarkers for the disease.

As a follow-up study, the present research aimed to evaluate microRNA expression in exosomes isolated from individual plasma samples of patients with pseudoexfoliation (PEX) glaucoma. We compared these findings to those of a control group within a single ethnic cohort of Korean individuals, utilizing RNA sequencing without pooling samples. Plasma has much more volume to extract exosomes than AH, which is 100 µL at most from a single patient. This enables an individual test for each patient. It is important to obtain separate results from each patient without pooling because that test method has the potential to be used as a diagnostic biomarker detection. Although AH may be more specific for eye diseases, not all patients undergo ophthalmic surgeries so that we can acquire AH for further analysis. It would be unethical to draw AH from a patient, which is an invasive procedure that should be performed in an aseptic operating room, if a patient was not undergoing planned ophthalmic surgery. Therefore, it is difficult to use AH to detect a diagnostic biomarker for a first-line method. Until recently, no studies have reported on the detection of microRNAs in exosomes isolated from the plasma of patients with pseudoexfoliation (PEX) glaucoma.

## 2. Materials and Methods

### 2.1. Ethics Statement

This study was performed according to the tenets of the Declaration of Helsinki for human research. This study was approved by the Institutional Review Board (IRB) of Gyeongsang National University Changwon Hospital, Gyeongsang National University, School of Medicine (IRB number: GNUCH-2019-06-001-002). Written informed consent was obtained from all subjects who participated in the current study.

### 2.2. Diagnosis of PEX Glaucoma

Participants were evaluated at the glaucoma clinic of Gyeongsang National University Changwon Hospital by a single glaucoma specialist (H-K. C.). The diagnosis of PEX glaucoma was established based on the presence of pseudoexfoliation material on the anterior lens capsule or along the pupillary margin following maximal pupil dilation, in conjunction with the following criteria: glaucomatous optic disk changes, an initial intraocular pressure (IOP) of at least 22 mmHg, visual field defects indicative of optic nerve damage, and the exclusion of other conditions that could lead to secondary glaucoma [7].

### 2.3. Patient Selection and Acquisition of Plasma Samples

Plasma samples were obtained from patients with PEX glaucoma or control subjects after acquiring written informed consent. Control subjects only had simple cataracts. They had no other ocular disorders. One of the same trained nurses obtained blood samples (10 mL each). All of the collected samples were fully anonymized before being transferred to the research laboratories. Clinical data, including sex, age, and other ocular comorbidities, were retrieved from electronic medical records in a completely anonymized manner to ensure patient confidentiality.

### 2.4. Isolation of Exosomes from Plasma Samples

For plasma defibrination, 4 μL of thrombin was added per 0.5 mL of plasma, achieving a final concentration of 5 U/mL. The mixture was incubated at room temperature for 5 min with gentle mixing by flicking the tube, followed by centrifugation at 10,000 rpm for 5 min using a standard microcentrifuge. Upon the formation of a visible fibrin pellet at the bottom of the tube, the supernatant (serum) was transferred to a clean tube.

Exosomes were then isolated using the ExoQuick-TC kit. The collected serum was centrifuged at 3000× *g* for 15 min at 4 °C. The resulting supernatant was mixed with an exosome precipitation solution at a ratio of 250 µL of serum to 63 µL of ExoQuick reagent and incubated at 4 °C for 30 min. The mixture was subsequently centrifuged at 1500× *g* for 30 min, leading to the formation of a beige or white exosomal pellet at the bottom of the tube. The supernatant was discarded, and the remaining pellet with residual solution underwent an additional centrifugation step at 1500× *g* for 5 min. The supernatant was removed again, and the exosomal pellet was resuspended in 100–500 µL of sterile PBS for collection.

### 2.5. Western Blot

To confirm the presence of tetraspanin in the exosome pellet, CD63 Western blot was performed. Proteins were extracted from harvested cells using RIPA lysis buffer (Thermo Fisher Scientific, Waltham, Massachusetts, U.S.A. #89900) supplemented with a protease inhibitor cocktail (Thermo Fisher Scientific, #78430). The total protein concentration of each cell lysate was determined using the Bradford assay, with bovine serum albumin used as the standard.

Equal amounts of protein lysates (45 µg) were loaded onto denaturing polyacrylamide gels and subsequently transferred to a nitrocellulose membrane. Immunoblotting was performed using primary antibodies against CD63 (1:250 dilution; #ab134045, Abcam, Cambridge, MA, USA) and GAPDH (1:1000 dilution; #ab8245, Abcam, Cambridge, MA, USA). Horseradish peroxidase (HRP)-conjugated secondary antibodies were applied, and immunoreactive bands were detected using an enhanced chemiluminescence (ECL) reaction (Thermo Fisher Scientific, #32109). Digital chemiluminescence images were acquired using a Fusion Solo system (Vilber, Marne-la-Vallée Cedex, France).

### 2.6. Library Preparation and RNA Sequencing

Libraries were constructed using the NEBNext Multiplex Small RNA Library Prep Kit (New England BioLabs, Ipswich, MA, USA) for both test RNAs and the controls, following the manufacturer’s protocol [20]. Briefly, 180 pg of total RNA from each plasma sample was ligated with 1 µg of adaptors. Complementary DNA (cDNA) synthesis was performed using reverse transcriptase and adaptor-specific primers, followed by PCR amplification of the libraries.

The libraries were purified using AMPure XP beads (Beckman Coulter, Pasadena, CA, USA) and the QIAquick PCR Purification Kit (Qiagen, Germany). The size distribution and yield of the small RNA libraries were assessed using an Agilent 2100 Bioanalyzer with a High-Sensitivity DNA Assay (Agilent Technologies, Santa Clara, California, USA). High-throughput sequencing was conducted on a NextSeq500 system (Illumina, San Diego, CA, USA) using single-end 75 sequencing.

### 2.7. Data Analysis

Sequence read mapping was performed using the Bowtie2 software tool to generate BAM files, containing the alignment data. Sequences of mature miRNAs were used as the reference for mapping. Read counts were then utilized to determine the expression levels of miRNAs. The read counts corresponding to mature miRNA sequences were extracted from the alignment files using Bedtools (v2.25.0) [21] and the Bioconductor EdgeR package, which was implemented in the R statistical programming language (R Development Core Team, 2011, version 3.2.2).

Quality control was performed by trimming sequences using the BBDuk tool, with Illumina TruSeq adapters employed and a Phred quality score threshold set at 20. To facilitate inter-sample comparisons, quantile normalization was applied. Data visualization was performed using ExDEGA v1.2.1.0 software (EBIOGEN, Inc., Seoul, Korea).

For the miRNA target study, DianaTools-mirPath v.3 (http://diana.imis.athena-innovation.gr/DianaTools/index.php?r=site/page&view=software) was employed. DianaTools, miRTarBase (http://mirtarbase.mbc.nctu.edu.tw/php/search.php), miRWalk 2.0. (http://zmf.umm.uni-heidelberg.de/apps/zmf/mirwalk2/), and TargetScan (http://www.targetscan.org/vert_72/) were used to predict the miRNA targets. Related KEGG (Kyoto Encyclopedia of Genes and Genomes) pathways were analyzed in accordance with previous studies from Kanehisa Laboratories.

### 2.8. Statistical Analysis

microRNA validation data are presented as mean ± standard error of the mean (SEM). An unpaired Student’s *t*-test (Prism 5; GraphPad Software, La Jolla, CA, USA) was used for the statistical analysis. A *p*-value of <0.05 was considered statistically significant. Enrichment *p*-values were adjusted for the false discovery rate (FDR) [22].

## 3. Results

### 3.1. Demographics and Baseline Characteristics of Subjects

Seven PEX glaucoma patients and seven control subjects participated in the final study. The demographic characteristics of the participants are summarized in Table 1. The mean age of the PEX glaucoma group (*n* = 7) was 72.8 ± 9.2 years, while the mean age of the control group (*n* = 7) was 64.4 ± 9.8 years. All participants had no ocular comorbidities other than uncomplicated cataracts.

### 3.2. Isolation of Exosomes from Plasma Samples of PEX Patients and Control Subjects

**Figure 1:**
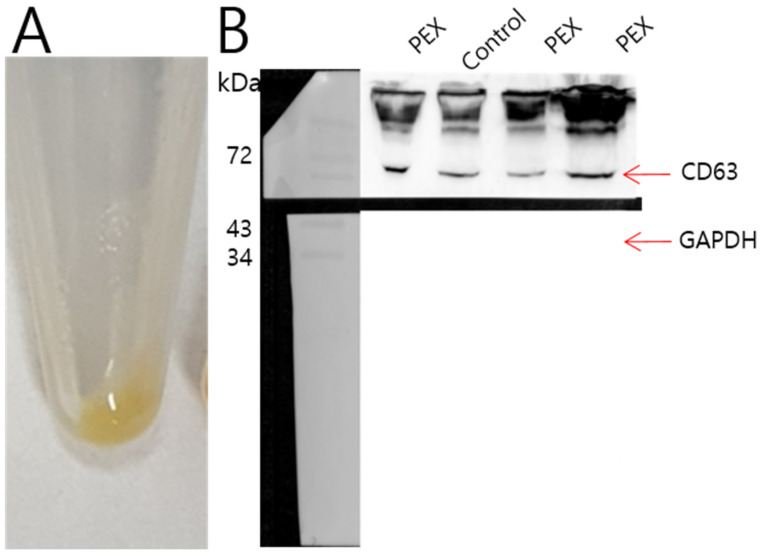
Isolation of exosomes from plasma samples of PEX patients and control subjects. Using plasma defibrinization and the Exoquick-TC kit, plasma samples collected from the PEX patients and control subjects were centrifuged to obtain exosomes as beige or white pellets at the bottom (**A**). To confirm that the exosome pellet contained tetraspanin, CD63 Western blot analysis was performed. Both exosomes from control subjects and PEX patients showed CD63 expression. However, PEX patients tend to have higher expression levels of CD63 than control subjects. GAPDH, the negative control for exosomes, showed complete negative expression in exosomes collected from both groups (**B**).

Plasma samples collected from PEX patients and control subjects were defibrinated to obtain supernatants (sera). Using an Exoquick-TC kit, the collected sera were centrifuged. Each supernatant was mixed with an exosome precipitation solution, incubated 30 min for serum at 4 °C, and centrifuged to obtain exosomes with beige or white pellet at the bottom (Figure 1A). The exosome pellet was collected and resuspended using phosphate-buffered saline (PBS). To confirm that the exosome pellet contained tetraspanin, CD63 Western blot analysis was performed. Both exosomes from the control and PEX patients showed CD63 expression. However, PEX patients tended to have higher expression levels of CD63 than control subjects. GAPDH, a negative control for exosomes, showed complete negative expression in exosomes collected from control subjects or PEX patients (Figure 1B and Figure S1).

### 3.3. Differential Exosomal miRNA Expression in Plasma Samples from Pseudoexfoliation Glaucoma Patients

**Figure 2:**
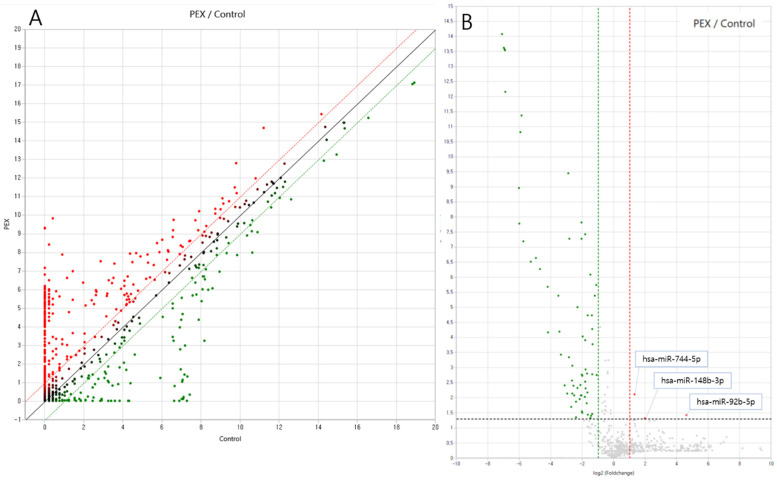
Scatter and volcano plot of miRNA expression in plasma samples from pseudoexfoliation glaucoma patients compared to the controls. (**A**) Upregulated miRNAs are visualized in the upper region of the scatter plot (red), and downregulated miRNAs are visualized in the lower region of the scatter plot (green). (**B**) In the volcano plot, upregulated miRNAs are shown in the right of the plot (red). Downregulated miRNAs are visualized in the left of the plot (green). The volcano plot shows three significantly upregulated miRNAs (hsa-miR-92b-5p, hsa-miR-744-5p, hsa-miR-148b-3p) compared to the control with fold change > 2 and *p*-value < 0.05.

miRNA targets were identified and analyzed using data from miRWalk 2.0. A total of 2588 miRNAs were examined through RNA sequencing. Among these, 330 mature miRNAs were detected within the exosomes of PEX glaucoma patients (Figure 2A). Upregulated miRNAs are shown in the right region of the plot (red), while downregulated miRNAs are displayed in the left region (green) (Figure 2B). Sixty-nine miRNAs demonstrated significantly differential expression, with a fold change >2 or <−2 and *p* < 0.05 in PEX glaucoma patients. Of these, three miRNAs were significantly upregulated compared to the controls: hsa-miR-92b-5p (fold change: 24.68), hsa-miR-744-5p (fold change: 2.49), and hsa-miR-148b-3p (fold change: 3.96). Additionally, 66 miRNAs were significantly downregulated in PEX glaucoma patients (Table 2).

### 3.4. Biological Interpretation of Differentially Expressed miRNAs

**Figure 3:**
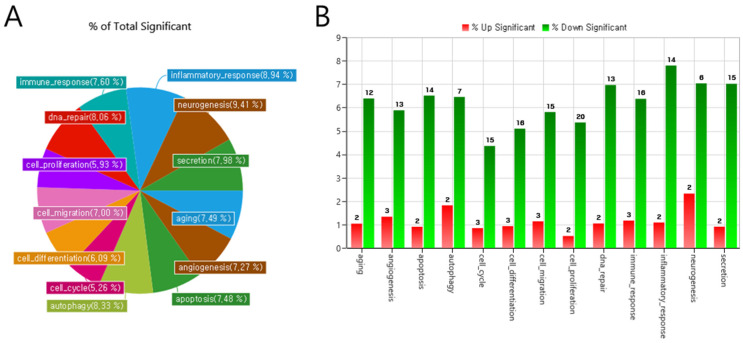
Percentage and number of microRNAs with significantly changed expression among gene ontology category-related microRNAs (pie, (**A**); bar, (**B**)). Gene ontology (GO) categories: (**A**) GO categories related to neurogenesis (9.41%) occupied the greatest portion, including both upregulation and downregulation. (**B**) Upregulated miRNAs are shown as a red graph and downregulated miRNAs are shown as a green graph.

Gene ontology (GO) analysis was conducted to evaluate the effects of the significantly differentially expressed miRNAs. From numerous GO pathways, fifteen major categories were randomly selected. In Figure 3, the percentage of genes with significant differential expression across each GO-related category is visualized. This percentage represents the proportion of miRNAs modified in PEX glaucoma relative to the total exosomal miRNAs identified in each category.

GO categories of miRNAs in exosomes with relatively large expression changes were identified in PEX glaucoma. GO categories related to neurogenesis (9.41%) had the greatest portion, including both upregulation and downregulation. Categories of inflammatory response (8.94%), DNA repair (8.60%), secretion (8.45%), and autophagy (8.33%) also presented significant proportions (Figure 3A).

Upregulated miRNAs are visualized as a red graph and downregulated miRNAs are visualized as a green graph. The GO categories of the three significantly upregulated microRNAs of the exosomes are involved in angiogenesis, cell cycle, cell differentiation, cell migration, cell proliferation, and immune response in PEX glaucoma patients. Significantly downregulated microRNAs of exosomes are shown in all GO categories (Figure 3B).

**Figure 4:**
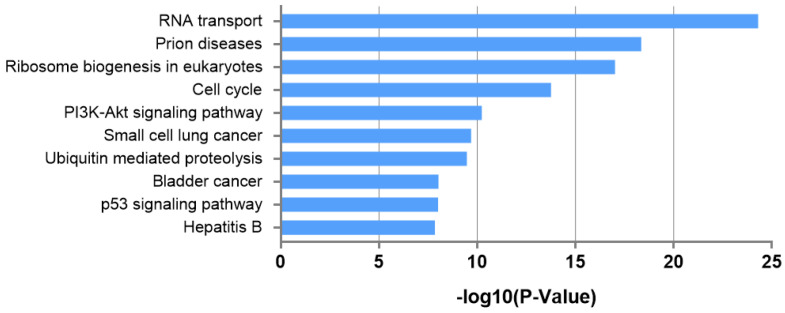
**KEGG pathway analysis** based on miRNA expression in plasma samples from pseudoexfoliation glaucoma patients compared to the controls. The *Y*-axis displays the top 10 most relevant pathways, ordered from the most to least significant. The *X*-axis shows the −log10-transformed *p*-values, indicating the statistical significance of each pathway.

The leading KEGG pathways, which include the predicted gene targets of each miRNA, are demonstrated in Table 3. The three most significantly enriched pathways, ranked by enrichment score (−log_10_ *p*-value), were RNA transport (24.30), prion diseases (18.35), and ribosome biogenesis in eukaryotes (17.02), respectively. Among these, the RNA transport pathway exhibited the most significant association with significantly differentially expressed miRNA from exosome in patients with PEX glaucoma in the KEGG pathway analysis (Figure 4).

## 4. Discussion

In this study, we assessed significantly differentially expressed miRNAs in exosomes derived from plasma samples of PEX glaucoma patients and controls using RNA sequencing in a single ethnic Korean group. A total of 3 upregulated and 66 downregulated miRNAs were identified in the exosome pellets of PEX glaucoma patients compared to the controls (Table 2). Among these, one upregulated miRNA (miR-744-5p) and two downregulated miRNAs (miR-1275 and miR-766) were found to possess an Exomotif, capable of its localization into the exosomes [23]. This suggests their possible roles in the exosomal cargo sorting mechanism and as a good diagnostic marker for patients with PEX glaucoma, even in patients who are not undergoing ophthalmic surgeries. Cargos of exosomes including protein, lipid, and RNA are sorted into exosomes by several means. Since the transfer of functional RNA-loaded vesicles may incorporate them into other cells to play a key role, the RNA contents of exosomes have been deeply investigated in different contexts [23]. Some miRNAs have a specific sequence motif (Exomotif) associated with the sumoylation of hnRNPA2B1, which regulates their sorting into exosomes [23]. We hypothesized that these miRNAs associated with Exomotif might have been loaded into exosomes during exosomal cargo sorting and winded up in the blood and eventually rejoined the venous system. Thus, they were detected in peripheral blood samples. However, not all microRNAs listed as significant in the result were selected. Since RNA cargos of exosomes are selected according to their specific sequences or other genes related to RNA sorting [24], further studies regarding the sorting and selection of cargo genes by exosomes are needed to clarify the overall mechanism.

Different eye disorders and different types of glaucoma might also influence the profile of exosomal miRNAs, as shown in previous studies; these findings further imply that exosomal miRNAs might serve as biomarkers for various eye diseases, including different types of glaucoma. A few previous studies have analyzed the miRNAs of exosomes in AH samples. Dismuke et al. have analyzed miRNA from exosomes extracted from the pooled AH of cataract patients during cataract surgery [25]. They reported that miR-486-5p, miR-204, and miR-184 were abundant among 10 different miRNAs. Gao et al. have analyzed the miRNA of exosomes from the pooled AH of diabetes and cataract patients [26]. They found that miR-551b was highly expressed compared with the age-related cataract group. Chen et al. have reported the expression profiles of the miRNAs of exosomes from the pooled AH of myopia patients [27]. They found five myopia-specific miRNAs: hsa-miR-582-3p, hsa-miR-17-5p, hsa-miR-450b-5p, hsa-miR-19b-3p, and hsa-miR-885-3p. These exosomal miRNAs were different from those found in the present study in PEX glaucoma patients, extracted from the exosomal miRNAs of plasma samples. These previous studies pooled AH samples to analyze exosomal microRNA without using individual AH samples [25,26,27]. Isolating exosome from the same AH sample and analyzing exosomal microRNAs at the same time without pooling is technically challenging. Instead, in this study, we used the plasma samples of these PEX glaucoma patients and control subjects to extract exosomes and perform analysis of the exosomal microRNAs in each sample without pooling. To our knowledge, this study is the first to evaluate significantly differentially expressed miRNAs in exosomes between PEX glaucoma patients and controls using individual plasma samples without pooling the samples.

A previous study by Hindle et al. [28] investigated miRNAs in both AH and plasma samples from PEX syndrome, or PEX glaucoma, POAG, and control subjects. They reported that at least 75% of miRNAs overlapped in both AH and plasma samples and were present from any one group (control or POAG/PEX glaucoma or PEX syndrome). However, they did not investigate exosomal miRNAs or exosomes from either AH or plasma sample. Since the lipid bilayer of exosomes protects cargos of genetic information from degradation, exosomal RNAs could be more stable than cellular RNAs [29]. This explains the superiority of exosomal miRNAs originating from the plasma as diagnostic biomarkers of PEX glaucoma with a minimally invasive procedure compared to other forms of cellular miRNAs detected from AH.

We conducted GO enrichment term analyses to identify the biological processes and molecular functions associated with the differentially expressed miRNAs. The results of GO analyses were mostly enriched in terms of being associated with neurogenesis (9.41%), inflammatory response (8.94%), DNA repair (8.60%), secretion (8.45%), and autophagy (8.33%). Among them, GO terms of neurogenesis, inflammatory response, and autophagy in PEX glaucoma were significantly related to the previous results of AH miRNAs in PEX glaucoma patients, although the exact same miRNA was not found between AH samples and plasma exosomes. The GO categories of neurogenesis and inflammatory response account for a major proportion in both PEX glaucoma and NTG in our previous study with AH samples of miRNAs [17,19]. Neurogenesis and inflammatory response also accounted for a substantial proportion in the present study in PEX glaucoma with plasma exosomal microRNAs. These biological processes appear to be significantly involved in the common pathogenesis of glaucoma. Autophagy dysfunction has been implicated in the pathogenic mechanisms of PEX syndrome at the cellular organelle level [12]. As a key process for maintaining cellular homeostasis, autophagy is crucial for the removal of protein aggregates [30]. Given the critical role played by autophagy in protein aggregates’ clearance, genetic variants in autophagy-related genes may contribute to the pathogenesis of PEX syndrome [31]. Studies have reported that Tenon’s cells from patients with PEX glaucoma exhibit distinct phenotypic alterations, including reduced autophagosome clearance due to autophagy dysfunction, compared to those from POAG patients [32]. The GO enrichment term “secretion” accounted for a proportion beyond negligence. Since secretion mentioned in the result is a GO term related to plasma exosome in glaucoma patients, it is considered logical to interpret it as “exosome secretion”. Hu et al. have suggested that the dysregulated secretion of exosomal miRNAs alters biological pathways, influencing the development of various cancers, including breast, colon, brain, and thyroid cancers [33,34,35], as well as neurodegenerative diseases such as Huntington’s and Parkinson’s disease [36,37,38]. Similarly, Ostenfeld et al. have demonstrated that the exosome-mediated secretion of tumor suppressor genes and miRNAs from bladder cancer cells can contribute to metastatic potential [39].

As revealed by the KEGG pathway analysis, the three most significant pathways were RNA transport (24.30), prion diseases (18.35), and ribosome biogenesis in eukaryotes (17.02), in the order of enrichment score. These molecular mechanisms suggest RNA transport and vesicle formation, which is highly relevant to our study results, that evaluated the microRNA from exosome, that is the extracellular vesicle. During the process of exosome biogenesis and secretion, extracellular vesicles are formed, accompanied by the transport of RNA, including miRNA. These KEGG pathway analysis results enhance the reliability of the present findings and suggest specific molecular mechanisms involving exosome-derived miRNAs.

This exploratory study is limited by its relatively small sample size. A further study comparing exosomal miRNAs in both AH and plasma samples is required to investigate the sorting mechanism and role of exosomal miRNAs in eye disorders, including glaucoma. However, it is meaningful that not only is the superiority of exosomal miRNAs more stable than cellular RNAs but also those of the peripheral blood samples could provide information regarding ocular tissues without a further invasive procedure, and they can provide the potential diagnostic marker of PEX glaucoma. Another limitation of this study is the age difference between the patient and control groups. Although both groups were elderly, the mean age of the PEX glaucoma patients was higher than that of the controls, which may have introduced age-related bias in the miRNA expression. Future studies with age-matched cohorts will be necessary to more accurately evaluate the impact of miRNA changes specific to PEX glaucoma. However, the age difference between the two groups may be attributed to the characteristic features of PEX glaucoma, which typically manifests at an older age compared to the controls or POAG patients [7]. This trend is consistent with the findings of the present study and has also been reported in previous research involving PEX glaucoma patients who underwent glaucoma surgery [40].

## 5. Conclusions

The exosomal miRNAs extracted from each plasma sample revealed significant differences in PEX glaucoma within a single Asian ethnic group (Korean), a comparison that has not been previously evaluated. These findings suggest potential roles for exosomal miRNAs in the pathogenic mechanisms of PEX glaucoma. Specifically, exosomal miRNAs, including hsa-miR-92b-5p, hsa-miR-744-5p, and hsa-miR-148b-3p, were significantly upregulated in PEX glaucoma patients compared to the controls. Upregulated miRNAs may serve as more promising diagnostic biomarkers than downregulated miRNAs for PEX glaucoma. Therefore, our results imply the potential of plasma exosomal miRNAs as biomarkers for glaucoma, particularly PEX glaucoma, in the diagnosis and prognosis of the disease. Further studies involving larger sample sizes and multicenter collaborations are needed to draw definitive conclusions.

## Figures and Tables

**Table 1:** Demographics of subjects.

Subject Number	Disease Status	Age, y	Sex	Ocular Comorbidity
1	PEX G	67	Male	None
2	PEX G	58	Male	None
3	PEX G	81	Female	None
4	PEX G	83	Male	None
5	PEX G	81	Male	None
6	PEX G	70	Male	None
7	PEX G	70	Female	None
8	Control	65	Female	None
9	Control	75	Male	None
10	Control	68	Female	None
11	Control	75	Male	None
12	Control	62	Female	None
13	Control	47	Male	None
14	Control	59	Male	None

PEX: pseudoexfoliation, G: glaucoma.

**Table 2:** Differentially expressed microRNAs from plasma exosome extraction in pseudoexfoliation glaucoma patients compared to control.

miRNA	Assay ID	Accession Number	Fold Change	*p*-Value	Expression Change
			**PEX G/Control**	**PEX G/Control**	
hsa-let-7b-5p	2	MIMAT0000063	0.284	0.000	Down
hsa-miR-16-5p	8	MIMAT0000069	0.25	0.002	Down
hsa-miR-20a-5p	14	MIMAT0000075	0.231	0.004	Down
hsa-miR-25-5p	20	MIMAT0004498	0.016	0.000	Down
hsa-miR-93-5p	32	MIMAT0000093	0.056	0.000	Down
hsa-miR-105-5p	43	MIMAT0000102	0.252	0.032	Down
hsa-miR-197-5p	48	MIMAT0022691	0.026	0.000	Down
hsa-miR-195-5p	130	MIMAT0000461	0.017	0.000	Down
hsa-miR-369-5p	160	MIMAT0001621	0.155	0.020	Down
hsa-miR-196b-5p	195	MIMAT0001080	0.28	0.010	Down
hsa-miR-451a	209	MI0001729	0.395	0.000	Down
hsa-miR-486-5p	218	MIMAT0002177	0.289	0.001	Down
hsa-miR-181d-5p	235	MIMAT0002821	0.007	0.000	Down
hsa-miR-532-5p	284	MIMAT0002888	0.088	0.000	Down
hsa-miR-92b-5p	295	MIMAT0004792	24.678	0.038	Up
hsa-miR-1224-5p	421	MIMAT0005458	0.092	0.000	Down
hsa-miR-744-5p	465	MIMAT0004945	2.494	0.008	Up
hsa-miR-1228-5p	505	MIMAT0005582	0.098	0.000	Down
hsa-miR-1275	576	MI0006415	0.192	0.014	Down
hsa-miR-1284	587	MI0006431	0.008	0.000	Down
hsa-miR-1538	605	MI0007259	0.19	0.045	Down
hsa-miR-3131	657	MI0014151	0.305	0.016	Down
hsa-miR-3158-5p	692	MIMAT0019211	0.236	0.008	Down
hsa-miR-3173-5p	707	MIMAT0019214	0.225	0.011	Down
hsa-miR-3605-5p	828	MIMAT0017981	0.244	0.008	Down
hsa-miR-3620-5p	843	MIMAT0022967	0.243	0.000	Down
hsa-miR-3652	852	MI0016052	0.163	0.007	Down
hsa-miR-3913-5p	901	MIMAT0018187	0.161	0.001	Down
hsa-miR-3925-5p	914	MIMAT0018200	0.14	0.000	Down
hsa-miR-4491	1038	MI0016853	0.287	0.000	Down
hsa-miR-4492	1039	MI0016854	0.38	0.000	Down
hsa-miR-4504	1052	MI0016867	0.32	0.000	Down
hsa-miR-4516	1065	MI0016882	0.159	0.003	Down
hsa-miR-4654	1125	MI0017282	0.315	0.007	Down
hsa-miR-4732-5p	1209	MIMAT0019855	0.367	0.041	Down
hsa-miR-6509-5p	1438	MIMAT0025474	0.009	0.000	Down
hsa-miR-6717-5p	1448	MIMAT0025846	0.388	0.000	Down
hsa-miR-6731-5p	1463	MIMAT0027363	0.016	0.000	Down
hsa-miR-6734-5p	1466	MIMAT0027369	0.246	0.029	Down
hsa-miR-6760-5p	1492	MIMAT0027420	0.241	0.000	Down
hsa-miR-6849-5p	1582	MIMAT0027598	0.019	0.000	Down
hsa-miR-7706	1658	MI0025242	0.248	0.002	Down
hsa-let-7d-3p	1728	MIMAT0004484	0.284	0.003	Down
hsa-miR-92a-3p	1753	MIMAT0000092	0.388	0.002	Down
hsa-miR-16-2-3p	1763	MIMAT0004518	0.321	0.037	Down
hsa-miR-128-3p	1813	MIMAT0000424	0.473	0.002	Down
hsa-miR-142-3p	1821	MIMAT0000434	0.016	0.000	Down
hsa-miR-106b-3p	1850	MIMAT0004672	0.358	0.048	Down
hsa-miR-30c-1-3p	1852	MIMAT0004674	0.116	0.004	Down
hsa-miR-148b-3p	1897	MIMAT0000759	3.962	0.048	Up
hsa-miR-486-3p	1921	MIMAT0004762	0.31	0.002	Down
hsa-miR-92b-3p	1983	MIMAT0003218	0.388	0.035	Down
hsa-miR-576-3p	1989	MIMAT0004796	0.129	0.007	Down
hsa-miR-766-3p	2039	MIMAT0003888	0.174	0.008	Down
hsa-miR-708-3p	2052	MIMAT0004927	0.033	0.000	Down
hsa-miR-1180-3p	2066	MIMAT0005825	0.171	0.004	Down
hsa-miR-1306-3p	2094	MIMAT0005950	0.039	0.000	Down
hsa-miR-3158-3p	2131	MIMAT0015032	0.291	0.005	Down
hsa-miR-3162-3p	2133	MIMAT0019213	0.143	0.000	Down
hsa-miR-3177-3p	2136	MIMAT0015054	0.054	0.000	Down
hsa-miR-3913-3p	2179	MIMAT0019225	0.205	0.005	Down
hsa-miR-4677-3p	2232	MIMAT0019761	0.008	0.000	Down
hsa-miR-6509-3p	2383	MIMAT0025475	0.008	0.000	Down
hsa-miR-6729-3p	2399	MIMAT0027360	0.204	0.000	Down
hsa-miR-6765-3p	2434	MIMAT0027431	0.467	0.000	Down
hsa-miR-6777-3p	2446	MIMAT0027455	0.359	0.000	Down
hsa-miR-6880-3p	2547	MIMAT0027661	0.137	0.000	Down
hsa-miR-6885-3p	2552	MIMAT0027671	0.437	0.000	Down
hsa-miR-7161-3p	2580	MIMAT0028233	0.256	0.000	Down

Fold change >2 or <0.5, *p*-value < 0.05 as significant criteria.

**Table 3:** Top 10 ranked KEGG pathways. From left to right, the columns indicate the 10 most relevant pathways, the *p*-values transformed as −log10, the number of predicted target genes, and the number of associated miRNAs.

KEGG Pathway	*p*-Value (−log10)	Genes Predicted as Target	Related miRNAs
RNA transport	24.30041521	48	4
Prion diseases	18.35492993	8	4
Ribosome biogenesis in eukaryotes	17.0221273	27	2
Cell cycle	13.76476278	33	8
PI3K-Akt signaling pathway	10.23418479	62	9
Small-cell lung cancer	9.689671903	23	8
Ubiquitin-mediated proteolysis	9.470397836	33	6
Bladder cancer	8.029735666	14	7
p53 signaling pathway	8.007213183	20	8
Hepatitis B	7.848689919	33	9

## Data Availability

The datasets used in the current study might be shared upon reasonable request to Hyun-kyung Cho, MD, PhD.

## Supplementary Materials

The following supporting information can be downloaded at: https://www.mdpi.com/article/10.3390/ijms26094244/s1.
