# Supplementary material for: Exosomal microRNA from Plasma in Patients with Pseudoexfoliation Glaucoma of Korea
Source: Int J Mol Sci. 2025 Apr 29;26(9):4244. doi: 10.3390/ijms26094244 (PMC12071745; doi:10.3390/ijms26094244)
Supplement: Supplementary file 1 [file ijms-26-04244-s001.zip › ijms-3569859-supplementary.pdf]

SUPPLEMENTAL INFORMATION

MANUSCRIPT TITLE: Exosomal microRNA from plasma in patients with pseudoexfoliation glaucoma of Korea

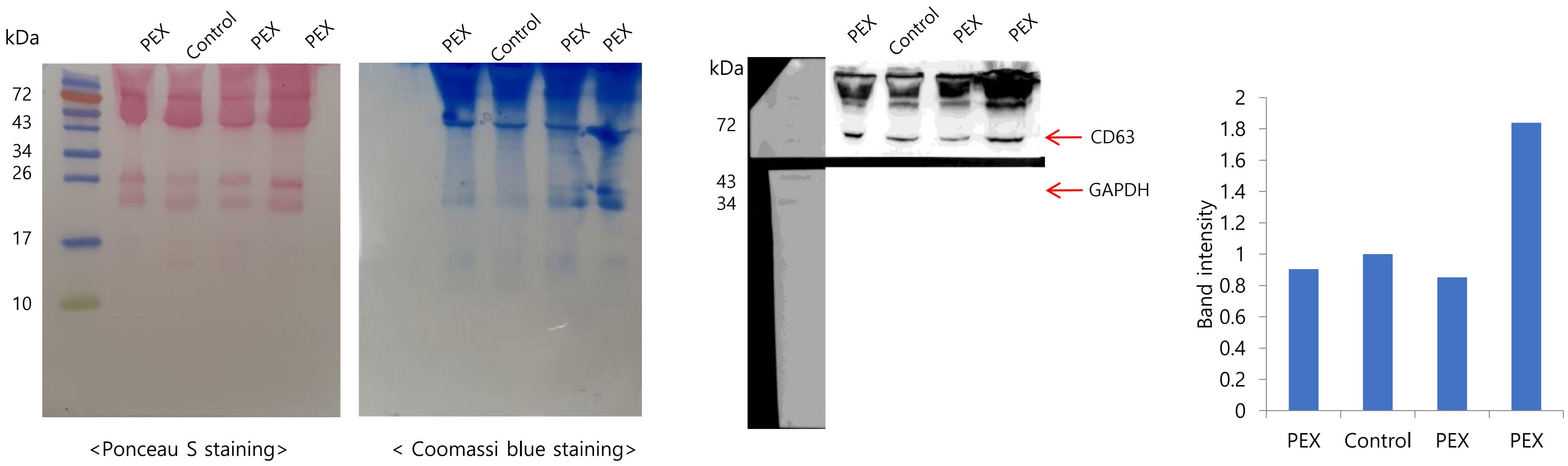

Figure S1.Original immunoblots used to crop the gel bands for Figure 1B. Quantitative analysis of band intensity for Figure 1B.
